# The future of software-controlled cooking

**DOI:** 10.1038/s41538-023-00182-6

**Published:** 2023-03-21

**Authors:** Jonathan David Blutinger, Christen Cupples Cooper, Shravan Karthik, Alissa Tsai, Noà Samarelli, Erika Storvick, Gabriel Seymour, Elise Liu, Yorán Meijers, Hod Lipson

**Affiliations:** 1 Department of Mechanical Engineering, Columbia University in the City of New York, 500 West 120th St., Mudd 220, New York, NY 10027 USA; 2 Department of Nutrition and Dietetics, Pace University, 861 Bedford Road, Pleasantville, NY 10570 USA; 3 Department of Food Technology, Wageningen University, 6708 PB Wageningen, Netherlands

**Keywords:** Science, technology and society, Engineering, Nutrition

## Abstract

To date, analog methods of cooking such as by grills, cooktops, stoves and microwaves have remained the world’s predominant cooking modalities. With the continual evolution of digital technologies, however, laser cooking and 3D food printing may present nutritious, convenient and cost-effective cooking opportunities. Food printing is an application of additive manufacturing that utilizes user-generated models to construct 3D shapes from edible food inks and laser cooking uses high-energy targeted light for high-resolution tailored heating. Using software to combine and cook ingredients allows a chef to more easily control the nutrient content of a meal, which could lead to healthier and more customized meals. With more emphasis on food safety following COVID-19, food prepared with less human handling may lower the risk of foodborne illness and disease transmission. Digital cooking technologies allow an end consumer to take more control of the macro and micro nutrients that they consume on a per meal basis and due to the rapid growth and potential benefits of 3D technology advancements, a 3D printer may become a staple home and industrial cooking device.

## Cooking in a digital world

Food printing is a process for producing physical, three-dimensional food products based on a computer model. Three-dimensional printing technology, which originally emerged in the 1980s^1^, was created to print different types of materials including plastic, metal, rubber, and concrete. However, the study of other potential uses is rapidly growing to include 3D printing of customized medicines^2^ and even human organs^3^. Today 3D food printing is still in its infancy, but may grow in popularity due to its customizability, convenience and other benefits that behoove the consumer.

Most of the cooking appliances currently in popular use, including cooktops, ovens, and microwaves, are analog devices requiring varying levels of manual involvement. These appliances also operate by heating an entire area by some uniform amount, which can lead to heating inefficiencies^4^. Over the past decade there has been an insurgence of devices that automate various cooking and preparatory kitchen tasks through the use of software; one of which is cooking via laser.

The heating type and resolution of laser cooking is most akin to broiling in the oven with the resolution of a creme brulee torch, respectively. Contrary to oven broiling, however, lasers can operate at various visible and non-visible wavelengths providing different cooking modalities^5,6^. As a means of comparison, lasers cook food by radiation heat transfer, microwaves also cook by radiation (by exciting water molecules), ovens cook food principally by convection (by circulating hot air)—this excludes broiling which uses infrared radiation—and foods are cooked on a stovetop via conduction (heat from a pan)^7^. Heat from a laser can also be modulated to a much higher degree than other heating appliances by adjusting the power, speed, wavelength, and beam intensity. Being that lasers are a fairly new application in the food domain^8^, more formal regulations would need to be implemented for more widespread use.

Though laser cooking can function as a standalone technology, its particularly well-suited for food additive manufacturing (AM) because of its high resolution and penetrative heat qualities^5^. AM in food production began in 2007^9^ and has since been explored by academia^10^ and industry^11–14^. The first commercially available chocolate printer was launched in 2012^15^ and NASA has explored the printing of food for space travel^16^. Food printing involves a roboticized system that deposits food pastes, powders, and liquids in a precise spatial arrangement, according to a digital blueprint. Aside from a handful of companies in the food printing space, other innovators and chefs alike are developing bread-making bots^17^, salad assembly machines^18^, pizza-making robots^19,20^, plant-based meat 3D-printers^21^, multi-ingredient food assembly machines^10^, pasta printers^22^, automated cake decorators^23^, personalized vitamin gummies^24^, and other software-controlled heating appliances^25–28^.

Many commonly consumed foods in the grocery store underwent some type of extrusion during their manufacturing process. “Printing food” is merely the controlled deposition of an ingredient; as such, any ingredient that was extruded as a paste (e.g. peanut butter, Nutella, vegetable puree, mustard, ground beef, sausage, chicken nuggets) can be classified as “printed.” Moreover, ketchup or mustard on a burger or frosting on a cake also contain deposited—or printed—materials. Therefore, 3D printing can be facilitated by a person or a computer.

Additionally, automation is widely used for repetitive processes such as flipping burgers^29^ or spreading sauce on a pizza^30^. Machines—unlike people—don’t get tired and every action can be precisely and accurately controlled even after thousands of repetitions. While current software-integrated food-facing machines transition manual control away from the user, they also give the user more creative-control by off-loading the mental energy that would otherwise be used by a human worker to manipulate objects repetitively and precisely. Commercial assembly and cooking robots are effectively pre-programmed as pick and place machines. True innovation will come from robots that give chefs the direct ability to customize, design, assemble, and cook their meals using software techniques—a process that doesn’t currently exist commercially.

## Food printing in today’s landscape

Foods that are printed would be categorized as “processed” given that in the process of preparation a food must be altered—made into a paste—in order to make this cooking method work. Given a growing shift of consumer preferences away from processed and towards whole—rather than processed—foods, 3D food printing may seem anathema to today’s food trends. A recent emphasis on locally-grown whole foods suggests that the pendulum is swinging back to the nation’s turn of the 20th century diet that was based on affordable real foods, rather than manufactured food products. There is also a distinct consumer preference for “naturalness” in food products^31^.

Processed foods have consistently received criticism from health authorities such as the Academy of Nutrition and Dietetics and the World Health Organization. Processed foods arose from urbanization, industrialization and the marketing of processed convenience foods to the post World War II consumer^32^ and have led to an overweight and obesity crisis costing the U.S. $50 billion per year in compromised worker productivity and healthcare expenses^33^. Overweight and obesity are the primary underlying factors for heart disease, type 2 diabetes, several types of cancer, and other chronic conditions^34^.

Today, foods consumed as part of a typical Western diet depend upon culture, income, food, affordability, and availability. The 400,000 food items that exist on the retail market range from fresh, whole foods that are perceived as expensive, to easy-to-prepare, highly processed (or as more foods are classified today, “ultra-processed”) foods (HPFs) that are nutrient-poor and energy dense^35^. The latter normally contain added fat, sugar, and sodium that extend shelf life and maximize palatability. Processed foods leave behind a considerable carbon footprint, especially when packaged and shipped to market^36^.

Food “processing” includes a wider span of foods than most consumers realize. Steps as routine as chopping, blending or pureeing foods are considered processing methods^37^. The main purposes of food processing include improving taste and texture, killing pathogenic micro-organisms and extending shelf life. Processing foods can affect its nutrient content, since high levels of heat, light, or oxygen can have this effect^37^. Vitamins most vulnerable to loss during processing include some of the most important for human health: folate, thiamine and vitamin C^37^. Some foods’ nutrient content is actually improved by processing^37^. As the technology evolves, printing food will continue to improve to avoid nutrient degradation.

We also see other important uses for 3D food printing, including creating alternatives to bland, unattractive pureed foods for those with swallowing and other digestive disorders^38–40^. Bringing new textures and shapes to food can enliven its attractiveness while allowing for production on a large scale in a factory or foodservice kitchen setting in hospitals and other operations. The precision of ingredient types and amounts that 3D printed food offers may also be useful for those who must consume very precise quantities of macronutrients, such as those who must limit certain amino acids or nutrients due to particular medical conditions. Printed food may also serve an important role as a sanitary source of food during pandemics such as COVID-19.

Printing with food may also allow for considerable environmental sustainability. Ingredients could be sourced and processed for consumption locally, assisting local farmers and food purveyors. Advocates also point to this technology’s ability to help produce products such as plant-based meats^41^, algae, and lower-cost unconventional proteins^42^ to consumers. Printed and laser-cooked food also offers opportunities for manufacturers to extend shelf-life, since the heat, light and oxygen involved in the process can be controlled on a millimeter scale^5^. Lastly, food waste could also be reduced since users would just be printing the ingredients they want to consume.

Proponents claim that the food industry is constantly seeking innovation—food of different sizes, shapes and textures. Digital cooking in the form of food AM could fill this gap in the market, but it still requires further development to become efficient and intuitive for consumer use. Proponents of digital cooking claim that the shareability and the comprehensiveness of the technology should be the vision for the food of tomorrow.

Furthermore, those who advocate for AM in food production postulate that 3D printing may not further distance individuals from their food’s origins, but rather allow consumers to choose foods grown closer to home and customize them for their personal tastes, energy and nutritional needs. They posit that the technology takes much of the mental and physical labor out of cooking and lends itself to the enjoyment of at-home cooking. Research supports the notion that more frequent home cooking has been shown to lead to better health^43^. Printing also has the unique characteristic of uniting science, cooking, leisure, and art. The expected market size of this industry ($425 billion by 2025^44^) is a testament to the fact that interest in 3D food printing is growing.

## Barriers to adopting 3D-printed food

Although 3D food printing allows consumers to precisely calibrate the nutrient and calorie content of foods, the worldwide obesity crisis may continue to cast a dark shadow on processed foods. Based on research on highly processed foods (HPF), foods that contain little protein and fiber and made shelf-stable through added sugar, salt, and fat are thought to be potentially “addictive”^45^. This is because these foods are not filling and actually engineered to produce a “bliss point,” or the point at which taste, mouthfeel and factors like crunchiness are at the most desirable point for the average consumer^37^. Highly processed, unrefined “junk” foods tend to overstimulate the production of dopamine, which causes cravings^37,46^. Such foods also routinely contain phosphates, which can threaten the organs and bones^37,46^. Processed foods are also linked to chronic inflammation, which can lead to heart disease, dementia, neurological problems, respiratory problems, and cancer^46^. Printed food, which involves powders and pastes that result in nutrient degradation, may be similarly non-satisfying and conducive to the health problems mentioned above. On the other hand, even fruits and vegetables that are picked and unprocessed may suffer nutrient degradation during days or even weeks of transport for many miles, a process that also burns fossil fuels^47^.

Though nutrition science is continuing to expand, the U.S. and other parts of the world continue to battle an obesity and chronic disease epidemic^48^. Traditionally, nutrition recommendations were based on an epidemiological approach to understanding diet-disease relationships. This approach involved studying the health effects of individual nutrients and foods over time in mainly white population groups^49^. This approach has produced many associations, but few causal relationships between particular nutrients and diseases. A change in the way we think about nutrition, focusing on the synergies in whole foods rather than individual nutrients, may come about^50^.

Issues surrounding cost may affect consumers’ willingness to adopt 3D printers as a food preparation technique. Although 3D printers can be built to take up much less room in a kitchen—which is advantageous—the cost of purchasing one may be prohibitively high during early adoption. Companies may need to employ a “razor and blades” business model^51^ similar to that of Gillette and Nespresso where the printer would be sold at a low price and the reoccurring revenue stream would come from the purchase or subscription of food cartridges and recipe files. Another consideration may be how and at what temperature the food inks need to be stored. Limited cooking space and integration with other appliances can be a concern for many people, especially where space is paramount in more affluent city environments.

## Acceptance of 3D-printed food

The potential for widespread acceptance of 3D-printed food is difficult to determine at this early stage of development. Results of a dual period study in rapidly urbanizing China (1996–-2013) suggested that supply-side economics were not sufficient to predict consumer behavior in terms of processed food, eating out and convenience shopping. A complex set of attitudes, traditionalism and other factors impacted consumers’ choices^52^. Siegrist and Hartmann^53^ reported that this age of “disruptive technologies” demands an understanding of consumer motivations for trying new food technologies. This is particularly true because in recent studies, researchers found that naturalness of foods produced and trust in the industry producing a food technology are top factors that determine technology acceptance^53,54^. Technological attributes were found to be negative and natural attributes, positive. A negative image of highly processed food is strongly influenced by a preference for naturalness^53–55^.

They found that most individuals by nature tend to be conservative about new food technologies. Some factors influencing new technology acceptance are: degree of cultural dependence on them^53^. One approach to promoting 3D food printing is encouraging families to think about a 3D food printer in their home as a “mini food manufacturing plant” in that it can reduce food waste to zero, lower energy consumption and allow for recipe customization.

Limitations of current 3D food printers include the number of ingredients that can be used at a time and the ways to cook the food once ingredients are assembled. Precision cooking is the second crucial feature that has been lacking in current food printers. While printers give us the ability to deposit ingredients with millimeter precision, no commercial cooking device has the ability to heat with the same degree of control. Lack of precision heating limits these devices’ ability to print multi-material products such as meats and grain products that often require some form of targeted heating after ingredient deposition. Different foods require varying time and temperature exposures for optimal cooking. To address the challenge of precision cooking, lasers are under investigation as a viable cooking technology and have shown to be effective at palatably cooking various food products^5,6,56–58^.

From a practical standpoint, machines under development that can accommodate dozens of ingredients, will face the problems of recipe and ingredient availability. At the same time, there is no extant public repository of printable food ingredients or recipes for 3D food printing. This is akin to having an iPod with no MP3 music files to play. Supportive ecosystems may need to be developed to foster the growth of this technology: a repository of printable ingredients, a repository of digital recipes, a design software to model and optimize printable meals, and a supply chain for the manufacturing and dissemination of food printer cartridges. These food cartridges can consist of pastes (e.g. ground beef, peanut butter, Nutella), powders (e.g. paprika, chili powder, cumin), flakes (e.g. oregano, thyme, parsley), liquids (e.g. olive oil, vinegar, soy sauce), solids (e.g. salt, pepper), and other edible items that can be deposited in a controlled manner. We foresee a business ecosystem funded by printers, print cartridges, and digital recipes that creates a sustainable revenue stream for equipment manufacturers, food suppliers, and digital recipe developer “food artists” catering for a variety of convenience, nutrition, and cost preferences.

## A practical demonstration of digital cooking

**Fig. 1:**
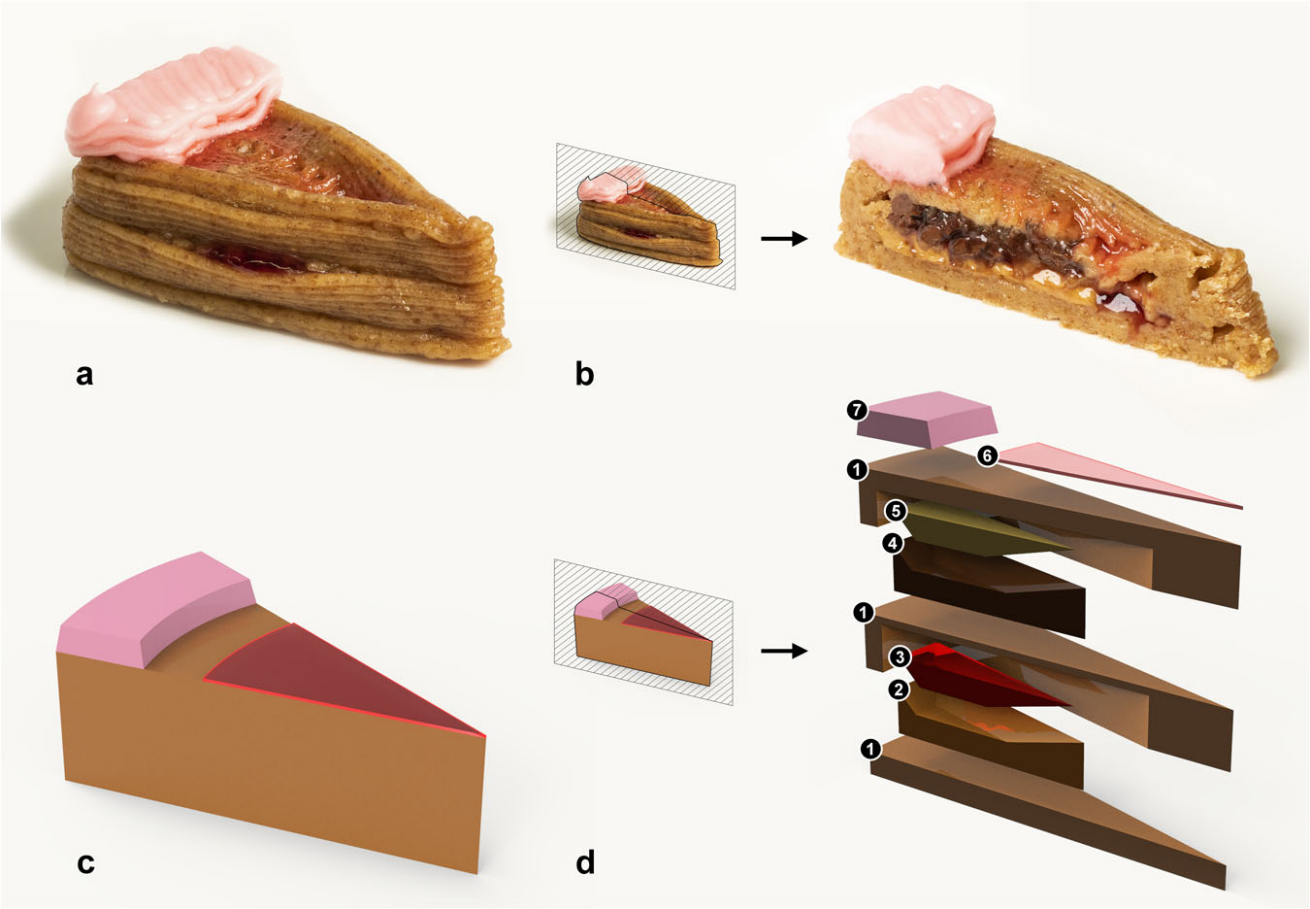
A seven-ingredient 3D-printed slice of cake. **a** The final printed food product (V7). **b** A cross-sectional cut of the final-printed slice showing internal ingredients. **c** A 3D model rendering of the final food product. **d** A cross-sectional view of the cake showing how each of the ingredients are layered. The ingredients that were used are as follows: (1) graham cracker paste, (2) peanut butter, (3) strawberry jam, (4) Nutella, (5) banana puree, (6) cherry drizzle, and (7) frosting.

As a demonstration of our digital cooking approach, we challenged ourselves to create a system that can combine many ingredients and cook them in-line. As a stretch goal, we attempted to print and laser-cook a seven-ingredient slice of cake (Fig. 1a), which, to our knowledge, is a record setting number of ingredients in a single printed food product (Supplementary Video 1, Supplementary Code 1). Our printing process is akin to fused-deposition modeling (FDM), which is more commonly associated with producing plastic parts, but other printing methods such as powder bed fusion^59^ and binder jetting^60^ also exist for food. Contrary to FDM, however, our machine can also thermally process deposited ingredients using diode lasers and our print nozzle is notably bigger at 1.5 mm inner outlet diameter (more details can be found in the Supplementary Materials). We used a blue laser (operating at 445 nm) and a near-infrared laser (operating at 980 nm) as precision heating appliances since they have emerged as a versatile cooking technology for thin-layered ingredients^6,56,57,61^ since the light they emit can be precisely targeted and controlled for custom cooking.

After seven design iterations, we successfully assembled and selectively cooked a seven-ingredient confectionery dessert entirely using software (i.e. without user intervention). Through this iterative design process we found that food materials need to be classified as “structural” or “filler” ingredients based on viscoelastic properties, such that they can be more accurately placed within a design model to eliminate failures due to printing (Supplementary Figure 1). These findings seem to match conventional intuition for conventionally assembled meals as well; cakes and layered foods tend to have more liquid fillers either atop or nestled within more structural grain-based ingredients (e.g. apple pie, cupcakes, and cheesecake).

With each successive print, our model needed to incorporate more structural ingredients to minimize print failures. Table 1 illustrates this point in material composition for each ingredient in our model. More structural ingredients such as graham cracker ended up becoming a foundational ingredient for each layer of the assembly while peanut butter and Nutella would act as supporting layers for less structural ingredients (also visible in Fig. 1d). The design of our print became similar to constructing a home where floors, walls, and ceilings being the foundation (graham cracker) and inner pools (Nutella and peanut butter) holding softer ingredients within (banana and jelly). Moreover, ingredients that exhibit a higher extrusion multiplier—the flow rate of an ingredient—also tend to be more viscous and make up a larger part of the final printed product.

**Table 1:** Material composition (% by volume) of each printed structure and success rate. Ingredients are listed from most structural to least structural (top to bottom row). Each column shows a different design iteration (from V1 to V7). The final print time was approximately 30 min.

Ingredient	V1	V2	V3	V4	V5	V6	V7
Graham cracker	32%	33%	33%	59%	64%	73%	71%
Peanut butter	16%	23%	27%	14%	12%	7%	8%
Frosting	4%	4%	4%	4%	4%	7%	7%
Nutella	16%	23%	27%	14%	12%	7%	8%
Jelly	16%	8%	4%	4%	4%	2%	3%
Banana	16%	8%	4%	4%	4%	2%	3%
Cherry	1%	1%	1%	1%	1%	1%	1%
Result	Failure	Failure	Failure	Failure	Failure	Success	Success

Constructing edible meals via AM—rather than by hand—gives us the ability to localize flavors and textures on a millimeter-scale to create new food experiences. In this print, we recreated a familiar looking slice of cake, but it didn’t need to be ordinary-looking. Controlling the extrusion path gives us the ability to create unique lattice structures and interwoven ingredient combinations that are otherwise impossible to recreate using conventional extrusion or molding methods^62^. Slightly more limiting than printing with plastic or metal, however, the complexity of deposited food ingredients is only limited by the rheology of the printed ingredients.

## Discussion

As digital cooking technologies become more ubiquitous, it is feasible that humankind will see the nutritional merits and drawbacks of having software-controlled assistants in the kitchen. 3D food printing has the potential to be the next frontier in cooking. Questions surrounding cost, ease of use and consumer acceptance will likely be top factors driving the trajectory of this technology. The spotlight shed on whole foods vs. processed foods for good health may influence consumers’ perception of this technology. However, with upcoming generations’ fascination with not only novel technologies, but also environmental sustainability and healthy eating, all of these are likely to influence the extent of adoption. Additionally, development of competing cooking technologies and advancements in nutrition science may come into play. An industry built around this technology may be on the horizon, creating a new vision of better nutrition, better food accessibility and palatability for many, increasing food safety and adding art and cutting-edge science to the most basic human need—nourishment.

## Methods

### Sample preparation

All ingredients were acquired from a local convenience store (Appletree Market, New York City, USA). The peanut butter (Skippy, Austin, USA), jam (The J.M. Smucker Company, Orrville, USA), Nutella (Ferrero SpA, Alba, Italy), frosting (Betty Crocker, Minneapolis, USA), and cherry drizzle (Krasdale Foods Inc, The Bronx, USA) required no additional processing prior to being packed into syringe barrels. We handmashed a banana with a fork until the consistency was uniform to ensure that the nozzle tip would not be obstructed during extrusion. To prepare the graham cracker paste, eight full sheets of graham crackers (140 g), 2tbs. of butter, and 4tsp. of water were combined and mixed in a Food Processor (Cuisinart, Stamford, USA) for less than a minute.

Each ingredient was packed into a syringe barrel (PN: 7012134), which was outfitted with a 14 gauge tapered nozzle tip (PN: 7018052) (Nordson EFD, East Providence, USA). The barrels were carefully packed with a spoon and the material was packed from the top of the barrel downward to avoid bubbles or air pockets, which could cause issues during printing. All ingredients were refrigerated prior to being packed into syringes for printing, this tended to thicken the ingredients and make them more structurally stable.

### Printing and cooking mechanism

We retrofitted an X-Carve Cartesian gantry (Inventables, Chicago, USA) with a custom extrusion mechanism (Supplementary Figure 2), allowing us to pick-and-place ingredients for printing. Our printer can accommodate up to seven ingredients, which are all housed on the front tool carriage (Supplementary Figure 3). We control the motion of all of the axes with ClearPath brushless servomotors (CPM-SDSK 2311S-RQN). The same system is used by Hertafeld et al.^10^.

Each ingredient cartridge consists of 30 mL syringe barrel (PN: 7012134) outfitted with a 14-gauge flexible tapered nozzle tip (PN: 7018052) placed in a custom 3D printed tool holder. These syringe tips have a 1.5 mm inner diameter at the food exit point, which results in a bead diameter of 1.5 mm for each deposited strand of food. An acrylic mounting plate was used to fixture the blue laser diode to the moving printer head. Supplementary Figure 4 shows the blue laser mounted to the extrusion mechanism on our gantry.

### Laser specs

Our cooking apparatus comprises a blue laser diode operating at 445 nm. At a current draw of 3 A, the maximum output power of this laser can be modulated to 13.8 W. For the experiments presented in this paper, we kept the current at 1.1–1.25 A, corresponding to a power output of approximately 5-6 W. Supplementary Table 1 presents more detailed specs on the laser spot size at various distances, as well as the divergence angle of the beam. Given the placement of the laser with respect to the food, the spot size of the laser was approximately 0.25 in.

### Designing meals

Solidworks (Dassault Systemes, Velizy-Villacoublay, France), a computer-aided design (CAD) software, was used to model our printed foods. Each material was modeled as a part file and then combined into an assembly prior to being exported for printing. Once fully modeled in CAD, parts were exported as an STL file, a standard stereolithography file format, allowing it to be processed by a slicer engine.

### Slicer engine

*Slic3r* is an open-source flexible toolchain that helps convert model representation files into G-code, a computer numerical control programming language, which can be interpreted by printer firmware. We optimized this existing software for our custom 3D printer. *Juli3nne*, our customized slicer engine, is a fork of the *Slic3r* project which tweaks the parameters of the slicer engine to enable printing of food material (code available in Supplementary Information).

Extrinsic and intrinsic parameters of the printer such as travel speed, in-fill density were adjusted to ensure the different food materials can be printed by modifying a single parameter—the extrusion multiplier. To determine the extrusion multiplier, each material is calibrated using a standard reference design. Once the extrusion multiplier is determined, each material and layer can be converted to its corresponding G-code (a.k.a. the digital recipe file). Supplementary Figure 5 provides an overview of the steps involved in generating the G-code for the 7-layer cheesecake print.

### Calibrating ingredients

To determine the extrusion multiplier associated with each material, a reference design of a cuboid of surface area 1 square inch was printed. The initial two layers of the cube are forced to be infill layers of the cube with an infill ratio of nearly 1. The rectilinear infill pattern helps determine the extrusion multiplier that needs to be set to ensure each pattern line doesn’t overlap with the previous printed line. By inspection, the extrusion multiplier is adjusted until the pattern is smooth and there are no smudges in-between layers.

Heuristically, the extrusion multiplier is set to 0.08 for materials with very high viscosity (e.g. graham cracker paste) and 0.03 for materials with low viscosity (e.g. jelly and banana puree). The viscosity is determined qualitatively; materials that have greater resistance to flow are assigned a higher extrusion multiplier. These values are constantly adjusted by a factor of 0.005 until no overlap in infill layers is observed. Supplementary Table 2 shows the variables that were used for the extrusion multiplier. Supplementary Figure 6 shows a sample of peanut butter that was calibrated using this method.

### Reporting summary

Further information on research design is available in the Nature Research Reporting Summary linked to this article.

## Supplementary information

Supplementary Materials

Reporting Summary

Supplementary Video 1

## Data Availability

The authors declare that all data supporting the findings of this study are available in the paper and supplementary information.

## Code availability

The digital recipe file that was used for the final printed structure can be found on github. *Juli3nne*, our slicer engine, can also be found on github; note that it requires Slic3r to run properly.
