# Supplementary material for: The future of software-controlled cooking
Source: NPJ Sci Food. 2023 Mar 21;7:6. doi: 10.1038/s41538-023-00182-6 (PMC10030630; doi:10.1038/s41538-023-00182-6)
Supplement: Supplementary file 1 — Supplementary Materials [file 41538_2023_182_MOESM1_ESM.pdf]

Supplementary Materials

The future of software-controlled cooking

Jonathan David Blutinger[1,*], Christen Cupples Cooper[2], Shravan Karthik[1], Alissa Tsai[1], Noa Samarelli[1], Erika Storvick[1], Gabriel Seymour[1], Elise Liu[1], Yorán Meijers[1,3], Hod Lipson[1]

Contents:

Descriptions………………………………………………………………………….... 2

Supplementary Figures S1 – S6…………………………………………………………….3

Supplementary Tables S1 – S2……………………………………………………………..9

[1] Creative Machines Laboratory, Mechanical Engineering, Columbia University, New York, NY 10027, USA.

[2] Department of Nutrition and Dietetics, Pace University, 861 Bedford Road, Pleasantville, NY, 10570, USA.

[3] Wageningen University, 6708 PB Wageningen, Netherlands

File name: Supplementary Materials

Description: Supplementary Figures and Tables.

File name: Supplementary Video 1

Description: Seven-ingredient printed dessert. This video provides a practical demonstration of a multi-ingredient print. We achieve a successful seven-ingredient print using an iterative design approach. Each multi-ingredient design was printed and iterated upon until a structurally stable print was achieved. Here, we illustrate our process and main experimental results.

Supplementary Figures

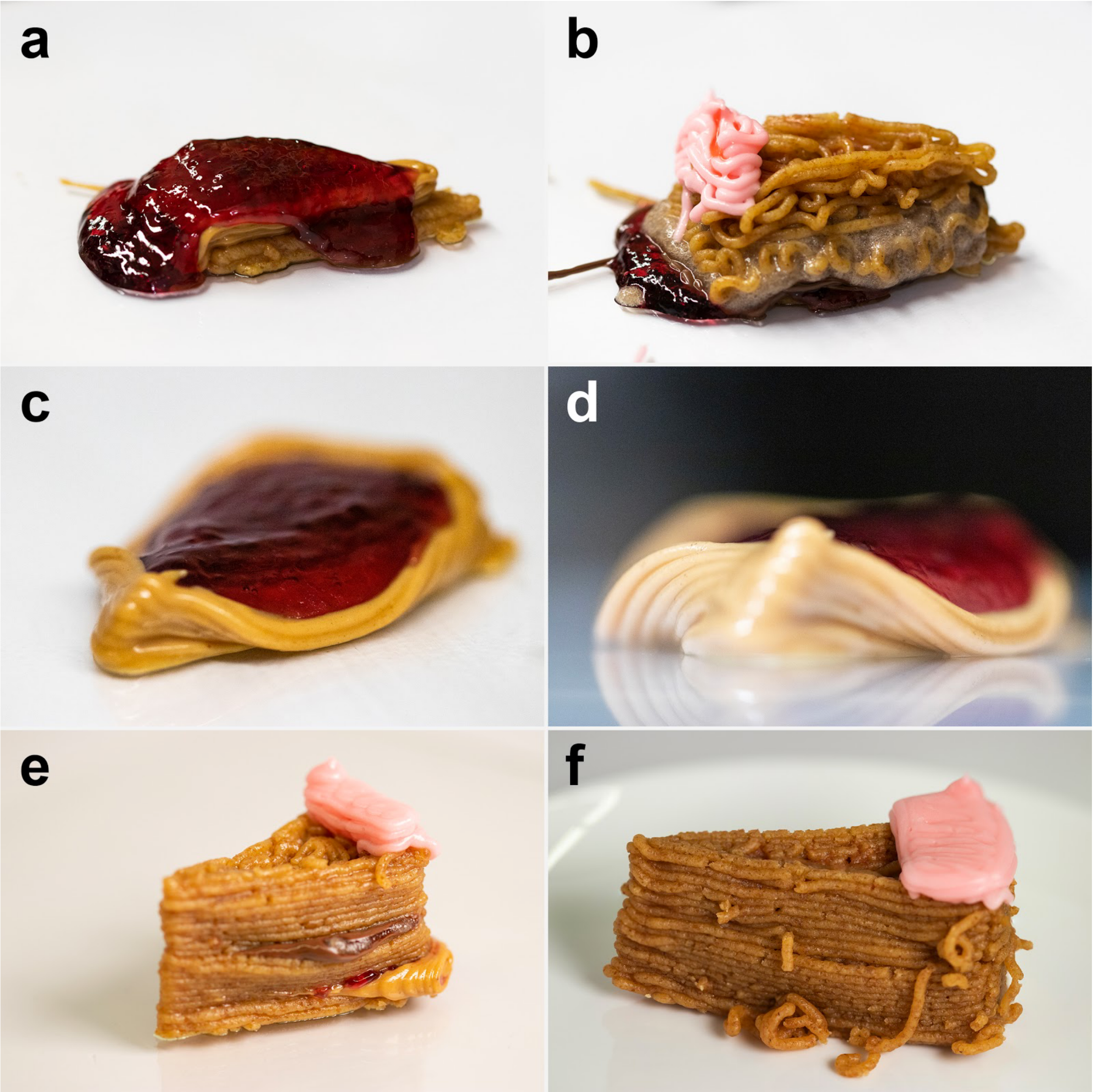

Supplementary Figure 1: *Failed cheesecake prints.* ***a*** *For initial designs, the jam could not hold its shape on a flat layer and needed to be pooled.* ***b*** *With no supporting walls, graham cracker paste crushed softer ingredients below it.* ***c***, ***d*** *Thin walls were prone to crumbling and needed to be tapered.* ***e*** *Adding graham cracker walls better supported the entire structure.* ***f*** *Occasionally, the graham cracker paste would form squiggles due to incorrect z-height or drying of the ingredient.*

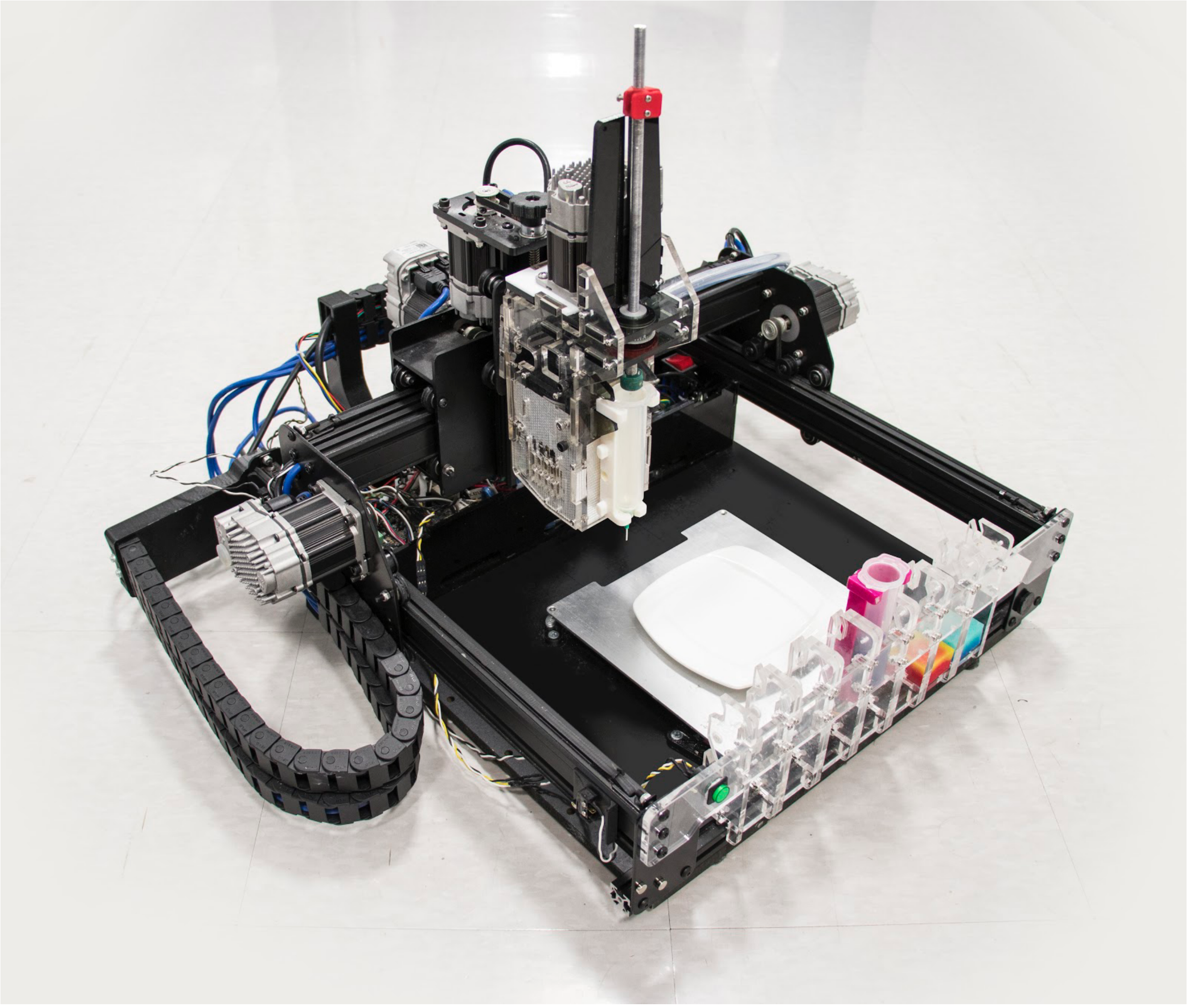

Supplementary Figure 2: *Our customized food printer. The extrusion mechanism and tool carriage were designed and attached to the body of the machine. Printer can accommodate up to seven ingredients.*

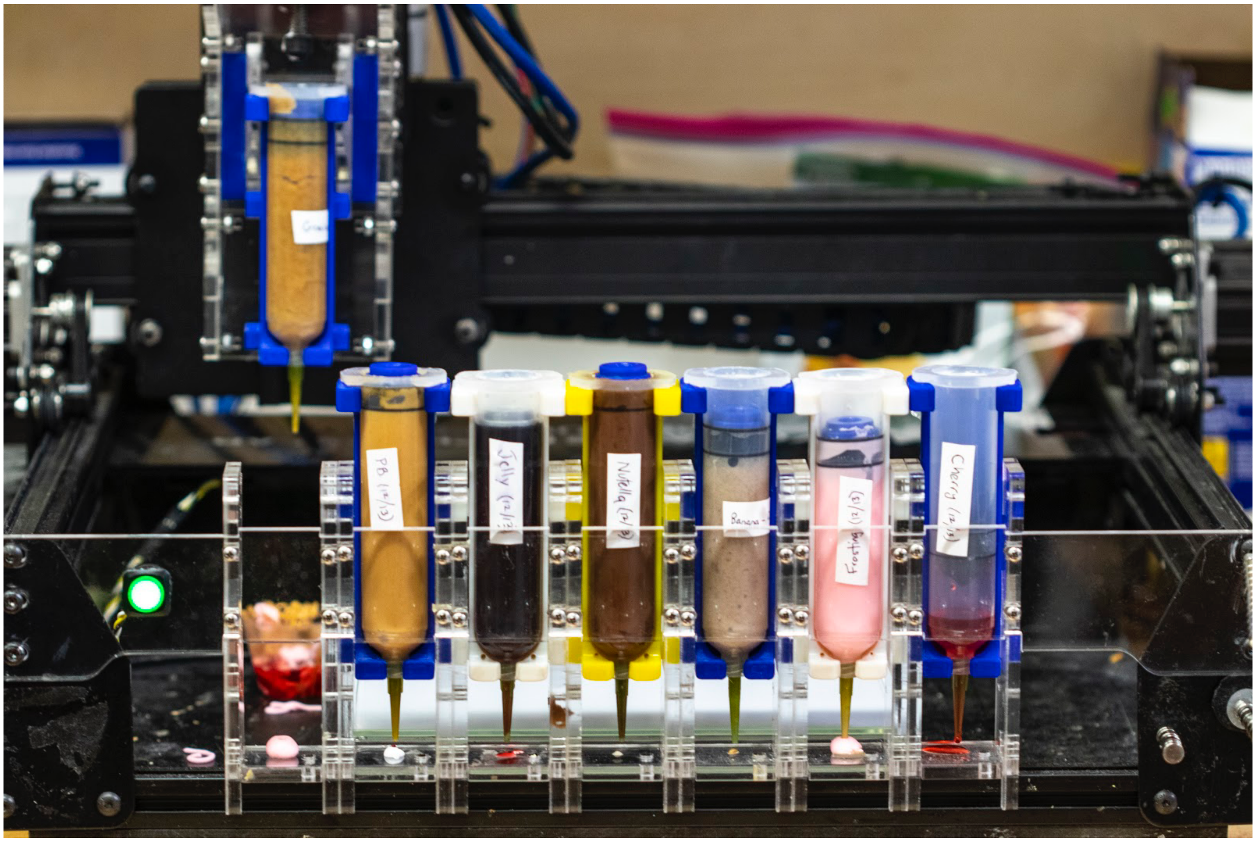

Supplementary Figure 3: *Front view of food printer showing seven filled food cartridges. Each cartridge has its own tool post and can be picked and placed by the extrusion mechanism.*

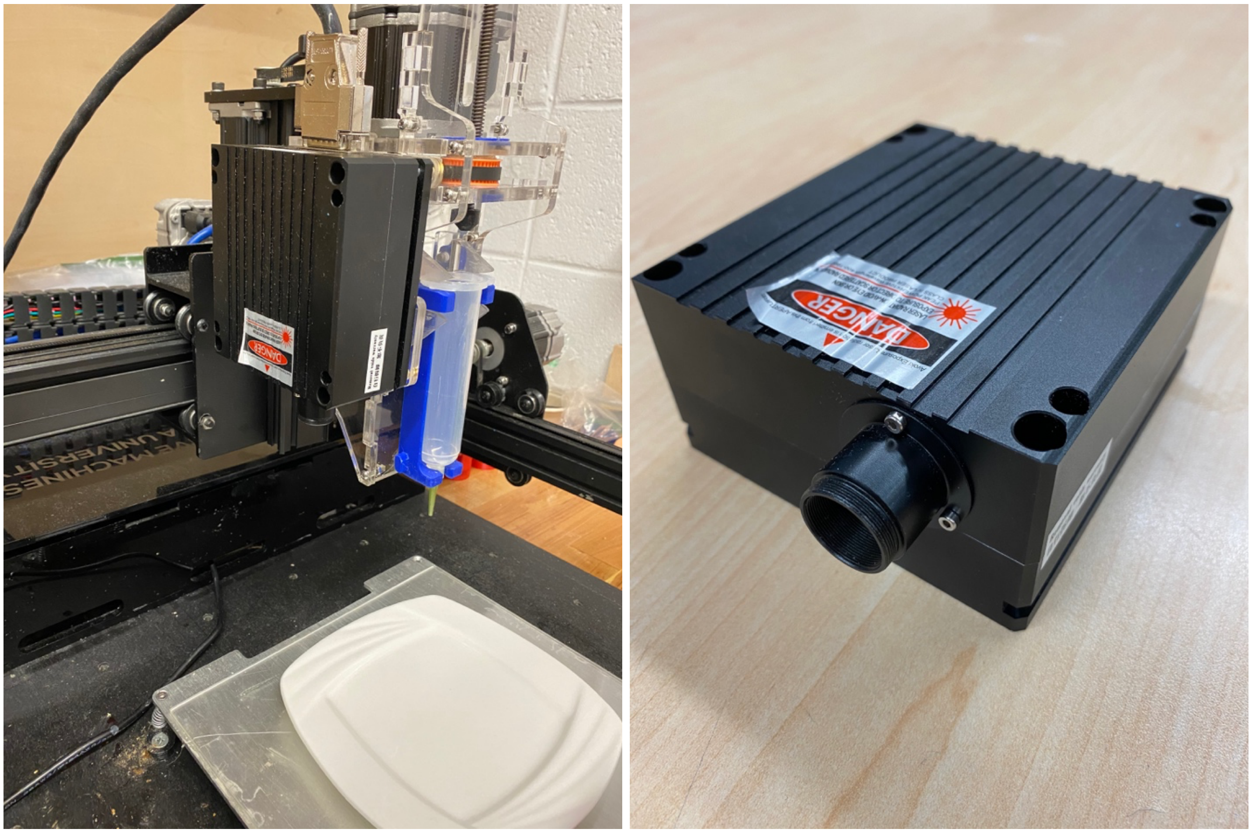

Supplementary Figure 4: *Isometric view of mounted laser (left) and close-up view of blue laser (right).*

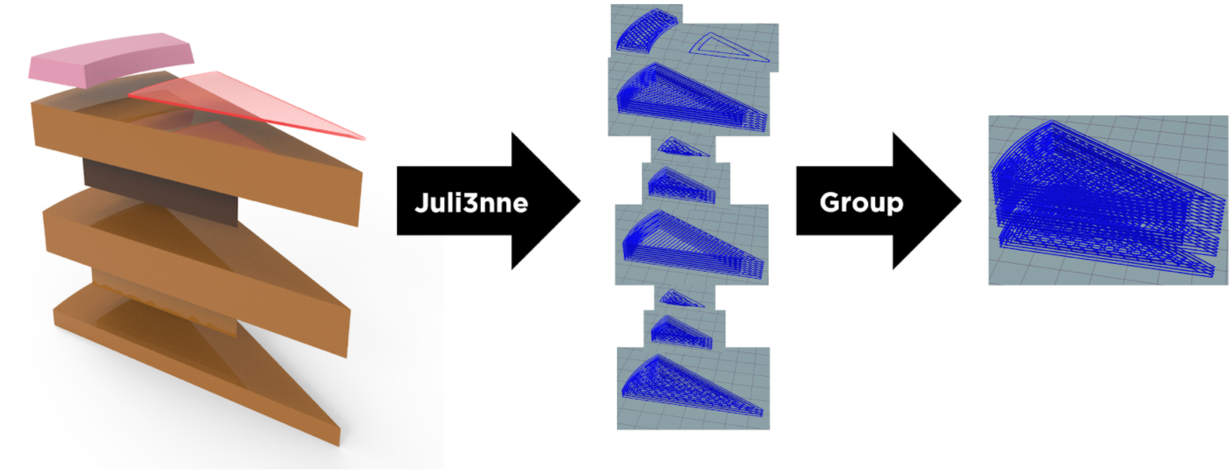

Supplementary Figure 5: *Generation of G-code from STL files using Juli3nne. Individual object files are sliced to layers and assembled to provide a single printable file.*

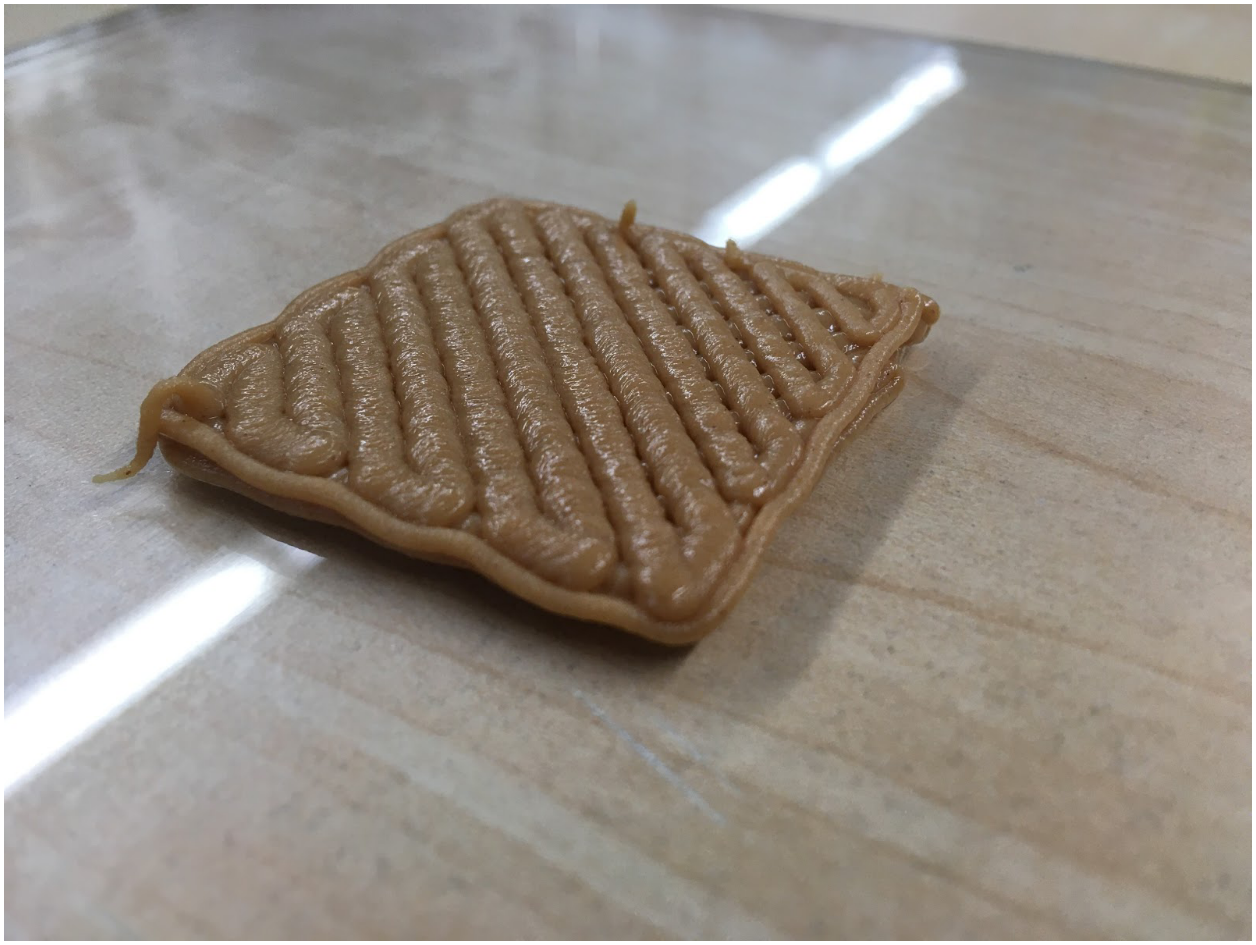

Supplementary Figure 6: *Calibration of peanut butter. The extrusion multiplier is adjusted until the rectilinear infill pattern has no overlapping sections.*

Supplementary Tables

	Horizontal	Vertical
**Beam size at aperture (mm)**	5.21	5.99
**Beam waist position (mm)**	0	0
**Beam waist size (mm)**	5.21	5.99
**Beam size at 4 m (mm)**	7.77	7.82
**Beam size at 14 m (mm)**	12.59	22.35
**Divergence (mRad)**	0.482	1.453

Supplementary Table 1: *Blue diode laser specifications.*

Ingredient	Extrusion multiplier
Graham cracker paste	0.100
Peanut butter	0.050
Cake frosting	0.045
Nutella	0.040
Banana puree	0.030
Strawberry jam	0.030
Cherry drizzle	0.005

Supplementary Table 2: *Extrusion multipliers used for ingredients in the printed cake.*
